# Factors Affecting the Efficacy and Safety of First-Line Anti-PD-1 Therapy in Advanced Non-Small Cell Lung Cancer

**DOI:** 10.32604/or.2026.079813

**Published:** 2026-06-16

**Authors:** Zhen Wang, Qian Xie, Yu Luo, Chuan Li, Wenqiang Guan, Yanling Zhang, Jidong Miao

**Affiliations:** 1 Department of Oncology, Zigong Fourth People’s Hospital, Ziliujing District, Zigong, China; 2 School of Education and Sports, Sichuan Vocational College of Health and Rehabilitation, Yantian District, Zigong, China; 3 Department of Critical Care, Qingdao Municipal Hospital, Shinan District, Qingdao, China; 4 Department of Respiratory and Critical Care Medicine, Qingdao Municipal Hospital, Qingdao University, Shinan District, Qingdao, China

**Keywords:** Advanced non-small cell lung cancer, PD-1 inhibitors, safety, immune-related adverse events, prognosis

## Abstract

**Objective:** This study assesses peripheral blood parameters as predictors of programmed cell death protein-1 (PD-1) inhibitor efficacy in advanced non-small cell lung cancer (NSCLC). **Methods:** We retrospectively analyzed 169 advanced NSCLC patients receiving first-line PD-1 inhibitor-based therapy. Baseline blood parameters and clinical characteristics were recorded. Logistic regression assessed associations with immune-related adverse events (irAEs). Chi-square tests compared efficacy and safety across treatment groups. **Results:** Baseline albumin/fibrinogen ratio (ALB/FIB) and PIV were associated with all-grade irAEs (*p* < 0.05), while PIV was markedly associated with grade ≥3 irAEs (*p* < 0.01). Multivariate analysis identified that the baseline pan-immune inflammation value (PIV) was independently associated with the occurrence of irAEs (*p* < 0.01). Compared to PD-1 inhibitor plus chemotherapy, adding bevacizumab increased oral mucositis (*p* = 0.010) and was linked to a later clinical stage (*p* = 0.001). In patients receiving peri-immunotherapy radiotherapy, leukopenia was more frequent (*p* = 0.030). **Conclusion:** Baseline PIV is independently associated with the occurrence of irAEs in advanced NSCLC patients receiving first-line PD-1 inhibitor therapy. Adding bevacizumab or radiotherapy may modify safety profiles.

## 1 Introduction

In 2022, there were 2.48 million newly diagnosed cases of lung cancer worldwide, with over 1.82 million deaths, making it the leading cause of both cancer incidence and mortality globally [1]. Non-small Cell Lung Cancer (NSCLC) accounts for approximately 85% of all newly diagnosed lung cancer cases [2]. Most NSCLC patients are diagnosed at an advanced stage, often accompanied by systemic metastasis [3]. Although conventional chemotherapy remains a primary treatment modality, it faces significant challenges, including limited efficacy, drug resistance, and severe side effects [4]. Studies have shown that the suppression of T-cell cytotoxic activity and the production of key cytokines, such as interleukin-2 (IL-2) and interferon-gamma (IFN-γ), facilitates tumor immune evasion and ultimately promotes tumor progression [5]. Programmed cell death protein 1 (PD-1) inhibitors, by blocking the interaction between PD-1 and programmed death-ligand 1 (PD-L1), significantly enhance T-cell cytotoxicity, offering new hope for the treatment of advanced NSCLC [6,7,8]. Moreover, the efficacy of these inhibitors is influenced not only by the intensity of the immune response but also by the occurrence of immune-related adverse events (irAEs) [9,10,11,12]. 

Inflammatory responses are closely associated with tumor initiation and progression. Inflammatory cells such as platelets (PLT), neutrophils (NEUT), and monocytes (MONO) contribute to tumorigenesis, invasion, and metastasis within the tumor microenvironment (TME) by upregulating cytokines and inflammatory mediators [13]. NEUT, MONO, and PLT can directly or indirectly influence the liver, facilitating the synthesis and release of C-reactive protein (CRP). Elevated CRP levels are positively correlated with the infiltration of CD8^+^ T cells and regulatory T cells [14]. Hyperactivation of effector T cells can trigger systemic inflammatory responses, increasing the risk of irAEs. 

Against this background, this study aims to predict the occurrence of irAEs in advanced NSCLC patients undergoing first-line PD-1 inhibitor therapy by monitoring peripheral inflammatory cell levels and calculating the baseline pan-immune inflammation value (PIV). This approach seeks to identify patients most likely to benefit from immunotherapy and optimize individualized treatment strategies. 

## 2 Material and Methods

### 2.1 Study Subjects

From November 2018 to October 2023, this study retrospectively collected data from 4450 patients with advanced NSCLC who were admitted to Qingdao Municipal Hospital for the first time. Through the application of inclusion and exclusion criteria, a total of 169 patients were ultimately selected, comprising 103 cases of adenocarcinoma and 66 cases of squamous cell carcinoma. All enrolled patients were negative for driver genes (wild-type). Inclusion and exclusion criteria are shown in the supplementary material (Fig. S1). 

These patients were divided into three groups: Group A consisted of 106 patients receiving PD-1 inhibitors combined with platinum-based doublet chemotherapy; Group B included 20 patients receiving PD-1 inhibitor monotherapy; and Group C comprised 43 patients receiving PD-1 inhibitors combined with bevacizumab and platinum-based doublet chemotherapy. Based on exposure factors, the entire study cohort was divided into two groups: the IT-RT group (receiving radiotherapy within 40 days before and after immunotherapy) and the IT-only group (receiving immunotherapy without radiotherapy). 

For patients who received radiotherapy (IT-RT subgroup), treatment was delivered using intensity-modulated radiotherapy (IMRT) or three-dimensional conformal radiotherapy (3D-CRT). Thoracic radiotherapy targeted the primary tumor or involved lymph nodes with doses ranging from 45 Gy to 66 Gy in conventional fractionation (1.8–2.0 Gy/fraction). Palliative radiotherapy for bone metastases typically employs hypofractionated schedules (e.g., 30 Gy/10 fractions or 20 Gy/5 fractions). Brain metastases were managed with whole-brain radiotherapy (30 Gy/10 fractions) or stereotactic radiosurgery (SRS) as appropriate. The decision to administer radiotherapy and the selection of target volumes and doses were at the discretion of the multidisciplinary team based on clinical indications. Radiotherapy was considered ‘within 40 days’ if it was initiated between 40 days before and 40 days after the first dose of immunotherapy, a window previously associated with optimal immune modulation. 

We confirmed that all methods were carried out in accordance with relevant guidelines and regulations. We confirmed that all experimental protocols were approved by the Ethics Committee of the Zigong Fourth People’s Hospital (Approval No.: 2024-LW-087-090). We confirmed that informed consent was obtained from all subjects. This study was conducted in accordance with the Declaration of Helsinki.

### 2.2 Data Collection

This study collected data on patients’ demographics, clinical characteristics, treatment outcomes, treatment-related adverse events (TRAEs), and irAEs. Within 7 days prior to the first dose of PD-1 inhibitor or bevacizumab administration, recent blood parameters were documented, including albumin (ALB), plasma fibrinogen (FIB), PLT, MONO, NEUT, and lymphocyte count (LYMPH). The PIV was then calculated as [(NEUT × PLT × MONO)/LYMPH], along with the albumin-to-fibrinogen ratio (ALB/FIB or AFR). 

### 2.3 Observation Indicators and Efficacy Evaluation Criteria

#### 2.3.1 Clinical Efficacy Evaluation

Following anti-tumor treatment, patients underwent imaging examinations every 1–3 cycles to assess changes in both pulmonary and extrapulmonary tumors. For patients receiving first-line PD-1 inhibitor therapy, clinical efficacy was evaluated according to the immune-related response criteria for solid tumors (iRECIST) [15]. Immune Complete Response (iCR) is defined as the disappearance of all target lesions, with pathological lymph nodes reduced to a short axis of less than 10 mm, and no new lesions appearing. Immune Partial Response (iPR) is characterized by a reduction in tumor size by 30% or more compared to the baseline. Immune-confirmed Progressive Disease (icPD) is identified by the emergence of new tumor lesions or an increase in tumor burden by 20% or more, confirmed upon reevaluation after one month. Immune Stable Disease falls between iPR and icPD. Progression-free survival (PFS) refers to the duration from the initiation of anti-PD-1 therapy in a patient to the occurrence of disease progression or death from any cause. Mean PFS was defined as the arithmetic mean of progression-free survival time for all patients. Median PFS (mPFS) represents the time point at which the cumulative progression-free survival rate reaches 50%.

#### 2.3.2 Adverse Reaction Grading

For patients receiving first-line PD-1 inhibitor therapy, from the initiation of PD-1 treatment until disease progression, irAEs were evaluated and recorded according to the Common Terminology Criteria for Adverse Events (CTCAE) version 5.0 and the NCI grading scale. The types, grades, and follow-up of irAEs were documented. All enrolled patients were classified according to the TRAE grading standard [16], ranging from grade I to grade V. Norris Drug Adverse Reaction Evaluation Criteria [17] is shown in supplementary material (Table S1). 

#### 2.3.3 Follow-Up

Patients were followed up through the hospital’s unified medical record system, PACS imaging system, and telephone communication. The follow-up period concluded in October 2023. Data collection included admission records, clinical course records, treatment plans, medical orders, laboratory results, and imaging reports. 

### 2.4 Statistical Analysis

Data analysis was conducted using SPSS version 26.0 (IBM Corp., Armonk, NY, USA) for statistical computations and GraphPad Prism version 9.0 (GraphPad Software, San Diego, CA, USA) for graphical representation. Categorical data were presented as frequencies (percentages), and differences in clinical characteristics between groups were assessed using chi-square tests or Fisher’s exact tests. Logistic regression analysis was employed to investigate the associations between various baseline factors and irAEs. During the selection process, variables with a *p*-value < 0.05 in univariate regression analysis were included in the multivariate analysis. Chi-square tests or Fisher’s exact tests were utilized to compare differences in clinical staging, recent efficacy assessments, one-year progression-free survival rates, and irAEs across groups.

## 3 Results

### 3.1 Baseline Characteristics

This study collected clinical baseline data from 169 patients with advanced NSCLC who received first-line PD-1 inhibitor therapy. Within the study cohort, NSCLC was classified as lung adenocarcinoma (61%) and squamous cell carcinoma (39%). Based on exposure factors, the cohort was divided into the IT-RT group (receiving radiotherapy within 40 days before or after immunotherapy) and the IT-only group (receiving only immunotherapy without radiotherapy). Details are provided in Table 1. 

**Table 1:** Clinical baseline characteristics of advanced non-small cell lung cancer (NSCLC) patients receiving first-line anti-PD-1 therapy.

Factors	Group A	Group B	Group C	IT-RT Group	IT-Only Group	*p*-Value
Age (year)						0.989
<65, n (%)	47 (44.3)	8 (40)	22 (51.2)	22 (47.8)	56 (45.5)	
≥65, n (%)	59 (55.7)	12 (60)	21 (48.8)	24 (52.2)	67 (54.5)	
Gender						0.759
Male, n (%)	95 (89.6)	13 (65)	34 (79.1)	37 (80.4)	104 (84.6)	
Female, n (%)	11 (10.4)	7 (35)	9 (20.9)	9 (19.6)	19 (15.4)	
Smoking history						0.358
Yes, n (%)	74 (69.8)	8 (40)	23 (53.5)	23 (50.0)	79 (64.2)	
No, n (%)	32 (30.2)	12 (60)	20 (46.5)	23 (50.0)	44 (35.8)	
Pathological type						0.294
Adenocarcinoma, n (%)	51 (48.1)	12 (60)	40 (93.0)	31 (67.4)	72 (58.5)	
Squamous cell carcinoma, n (%)	55 (51.9)	8 (40)	3 (7.0)	15 (32.6)	51 (41.5)	
Tumor staging						0.067
Stage III, n (%)	43 (40.6)	7 (35)	5 (11.6)	10 (21.7)	45 (36.6)	
Stage IV, n (%)	63 (59.4)	13 (65)	38 (88.4)	36 (78.3)	78 (63.4)	
Brain metastasis						<0.001***
Yes, n (%)	14 (13.2)	1 (5)	10 (23.3)	16 (34.8)	9 (7.3)	
No, n (%)	92 (86.8)	19 (95)	33 (76.7)	30 (65.2)	114 (92.7)	
Bone metastases						0.042*
Yes, n (%)	19 (17.9)	1 (5)	11 (25.6)	13 (28.3)	18 (14.6)	
No, n (%)	87 (82.1)	19 (95)	32 (74.4)	33 (71.7)	105 (85.4)	
Intrapulmonary metastasis						0.153
Yes, n (%)	65 (61.3)	6 (30)	24 (55.8)	24 (52.2)	79 (64.2)	
No, n (%)	41 (38.7)	14 (70)	19 (44.2)	22 (47.8)	44 (35.8)	
Surgery						0.030*
Yes, n (%)	30 (28.3)	5 (25)	7 (16.3)	6 (13.0)	36 (29.3)	
No, n (%)	76 (71.7)	15 (75)	36 (83.7)	40 (87.0)	87 (70.7)	
Programmed Death-Ligand 1 (PD-L1) expression level						0.715
<50%, n (%)	26 (24.5)	1 (5)	8 (18.6)	8 (17.4)	27 (22.0)	
≥50%, n (%)	22 (20.8)	16 (80)	5 (11.6)	11 (23.9)	32 (26.0)	
Unknown, n (%)	58 (54.7)	3 (15)	30 (69.8)	27 (58.7)	64 (52.0)	
mPFS						0.765
<mPFS, n (%)	53 (50.0)	10 (50)	21 (48.8)	22 (47.8)	62 (50.4)	
≥mPFS, n (%)	53 (50.0)	10 (50)	22 (51.2)	24 (52.2)	61 (49.6)	

Abbreviations: mPFS, median progression-free survival. *p < 0.05, ***p < 0.001, statistically significant difference.

### 3.2 The Diagnostic Value of Clinical Characteristics and Blood Parameters for irAEs

Univariate analysis revealed significant associations between irAE and factors such as intrapulmonary metastasis, tumor stage, ALB/FIB, and PIV. Multivariate analysis demonstrated that intrapulmonary metastasis (OR: 0.705, 95% CI: 0.106–4.690, *p* = 0.718), tumor stage (OR: 1.948, 95% CI: 0.970~3.913, *p* = 0.061), and AFR (OR: 1.180, 95% CI: 0.868–1.603, *p* = 0.291) were not independent predictors of irAE. However, PIV (OR: 1.014, 95% CI: 1.005–1.022, *p* = 0.002) was identified as an independent factor associated with irAE occurrence. Detailed results are presented in Table 2. 

**Table 2:** Univariate and multi-factor analysis of irAE in patients with advanced NSCLC.

Factors	Univariate Analysis			Multi-Factor Analysis		
	OR	95% CI	*p*-Value	OR	95% CI	*p*-Value
Gender						
Male (reference) vs. Female	1.653	0.708~3.857	0.245			
Age						
≥65 (reference) vs. < 65	1.277	0.696~2.343	0.430			
Smoking history						
Yes (reference) vs. No	1.030	0.553~1.920	0.925			
Brain metastasis						
Yes (reference) vs. No	1.032	0.441~2.414	0.943			
Bone metastases						
Yes (reference) vs. No	1.307	0.594~2.872	0.506			
Intrapulmonary metastasis						
Yes (reference) vs. No	0.532	0.283~0.999	0.047*	0.705	0.106~4.690	0.718
Tumor stage						
Stage III (reference) vs. Stage IV	2.181	1.12~4.247	0.022*	1.948	0.970~3.913	0.061
Surgery						
Yes (reference) vs. No	1.651	0.809~3.369	0.168			
Radiotherapy						
Yes (reference) vs. No	0.864	0.438~1.701	0.672			
AFR	0.912	0.835~0.998	0.044*	1.180	0.868~1.603	0.291
CAR	1.256	0.769~2.051	0.362			
PIV	1.012	1.004~1.019	0.003**	1.014	1.005~1.022	0.002**
NLR	1.058	0.942~1.189	0.339			
PLR	1.003	0.999~1.006	0.112			
LMR	1.001	0.82~1.223	0.989			

Abbreviations: ALB/FIB (AFR), albumin/fibrinogen ratio; CRP/ALB (CAR), C-reactive protein/albumin ratio; (NEUT × PLT × MONO)/LYMPH (PIV), (neutrophils × platelets × monocytes)/lymphocytes ratio, also known as the pan-immune-inflammation value; NEUT/LYMPH (NLR), neutrophils/lymphocytes ratio; PLT/LYMPH (PLR), platelets/lymphocytes ratio; LYMPH/MONO. (LMR), lymphocytes/monocytes ratio; OR, Odds Ratio; CI, Confidence Interval. *p < 0.05 indicates statistical significance. **p < 0.01.

### 3.3 The Incidence of irAEs under Different Treatment Modalities

Among the 169 patients included in the data analysis, 80 patients experienced irAEs of varying grades, with an overall incidence rate of 47.34% (80/169). As shown in Table S2, the most common irAEs were irAE dermatitis 15.38% (36/169), irAE thyroid dysfunction 9.47% (16/169), irAE pneumonitis 7.69% (13/169), and irAE gastrointestinal reactions 4.73% (8/169). Among these, ≥grade 3 irAEs accounted for 22.94% of all irAEs. The most frequent ≥ grade 3 irAE was irAE dermatitis 5.92% (10/169). The immunotherapy process was relatively stable, with a discontinuation rate of 13.02% (22/169) among all enrolled patients. The discontinuation rate in group A was 15.09% (16/106), in group C was 13.95% (6/43)], and there were no discontinuation cases in group B. Among all enrolled patients, 22 discontinued treatment due to severe irAEs. Clinical treatment decisions were made based on the type and grade of irAEs, with most irAEs improving after discontinuation of therapy, steroids, and symptomatic treatment. However, in group A, two patients (1.89%) with grade V irAE pneumonitis did not improve despite steroid and symptomatic treatment and died due to the severity of their underlying condition. 

### 3.4 The Predictive Value of Baseline Inflammatory Markers for irAE Occurrence Based on ROC Curve Analysis

**Figure 1:**
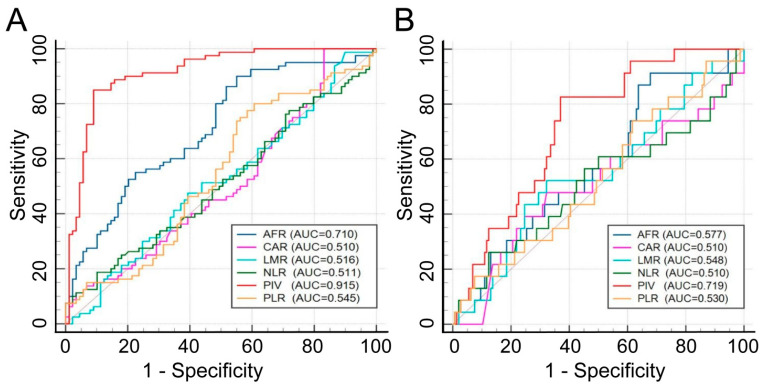
Predictive value of haematological indicators. (**A**) Receiver operating characteristic curves plotted according to the incidence of various grades of irAEs. (**B**) Receiver operating characteristic (ROC) curves plotted based on the incidence of grade ≥ 3 irAEs. AUC, Area under the curve.

Baseline inflammatory marker ROC curves were plotted based on the incidence of irAEs in patients (Fig. 1). When assessing the occurrence of irAEs in patients, the AUC for AFR was 0.710, with a sensitivity of 96.3%, specificity of 4.5%, and a cut-off value of 5.075. For PIV, the AUC was 0.915, with a sensitivity of 85%, specificity of 91%, and a cut-off value of 500.437. When evaluating the occurrence of grade ≥3 irAEs, the AUC for PIV was 0.719, with a sensitivity of 82.6%, specificity of 63%, and a cut-off value of 507.81 (Tables S3 and S4). 

### 3.5 The Impact of Different Combination Therapies on the Incidence of irAEs

In the comparison of various grades of irAEs, Group C patients exhibited a significantly higher incidence of oral mucositis (*p* = 0.010), with no other statistically significant differences observed (Table S5). In the IT-RT group, patients showed a significantly higher incidence of leukopenia (*p* = 0.030), with no other statistically significant differences noted (Table 3). 

**Table 3:** Comparison of the incidence and severity of irAEs between the IT-RT group and the IT-only group.

IrAE	All Patients	IT-RT Group	IT-Only Group	Statistical Values	*p*-Value
	*n* = 169	*n* = 46	*n* = 113		
Various grades of irAEs Dermatitis, n (%)	26 (15.38)	8 (17.39)	18 (15.93)	0.196	0.658
Thyroid dysfunction, n (%)	15 (8.88)	4 (8.70)	11 (9.73)	0.044	0.834
Gastrointestinal reactions, n (%)	8 (4.73)	4 (8.70)	4 (3.54)	3.853	0.051
Pneumonia, n (%)	13 (7.69)	2 (4.348)	11 (9.73)	0.996	0.318
Capillary proliferation, n (%)	9 (5.33)	3 (6.52)	6 (5.31)	0.179	0.672
Liver dysfunction, n (%)	3 (1.78)	1 (2.17)	2 (1.77)	1.073	0.300
Fever, n (%)	7 (4.14)	1 (2.17)	6 (5.31)	0.35	0.554
Neutropenia, n (%)	4 (2.37)	2 (4.35)	2 (1.77)	1.073	0.300
Oral mucositis, n (%)	5 (2.96)	1 (2.17)	4 (3.54)	0.136	0.713
Leukopenia, n (%)	4 (2.37)	3 (6.52)	1 (0.88)	4.722	0.030*
Anemia, n (%)	4 (2.37)	2 (4.35)	2 (1.77)	1.073	0.300
Blurred vision, n (%)	3 (1.78)	1 (2.17)	2 (1.77)	0.058	0.810
Myocarditis, n (%)	3 (1.78)	0 (0.00)	3 (2.65)	1.142	0.285
Fatigue, n (%)	2 (1.18)	0 (0.00)	2 (1.77)	0.757	0.384
Thrombocytopenia, n (%)	2 (1.18)	1 (2.17)	1 (0.88)	0.530	0.467
Dysphagia, n (%)	2 (1.18)	1 (2.17)	1 (0.88)	0.530	0.467
Telangiectasia, n (%)	1 (0.59)	0 (0.00)	1 (0.88)	0.376	0.540
IrAE severity					
Any grade, n (%)	111 (65.68)	34 (73.91)	77 (68.14)	0.18	0.672
Grade 1–2, n (%)	88 (52.07)	27 (58.70)	61 (53.98)	0.031	0.859
Grade ≥ 3, n (%)	23 (13.61)	7 (15.22)	16 (14.16)	0.139	0.709

*p < 0.05.

### 3.6 The Impact of Different Combination Therapies on Patient Prognosis

Group A patients: mPFS of 11.62 months, Mean PFS of 13.17 months, and a one-year PFS rate of 47.17%. Group C patients: mPFS of 10.87 months, mean PFS of 13.60 months, and a one-year PFS rate of 60.47%. As indicated in Table S6, Group C patients have a significantly later average clinical stage compared to Group A, with a statistically significant difference (*p* = 0.001). However, there is no significant statistical difference in recent clinical efficacy assessments between the two groups (*p* > 0.05). 

Group A (Stage IV) patients: mPFS of 9.90 months, mean PFS of 11.88 months, and a one-year PFS rate of 38.10%. Group C (Stage IV) patients: mPFS of 10.47 months, mean PFS of 12.92 months, and a one-year PFS rate of 42.11%. As indicated in Table S7, there is no significant statistical difference in recent clinical efficacy assessments between Group A (Stage IV) and Group C (Stage IV) patients (*p* > 0.05). 

### 3.7 Impact of Combined Radiotherapy and Immunotherapy on Patient Prognosis

**Figure 2:**
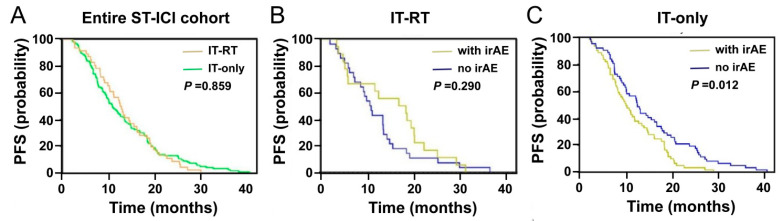
Prognostic differences between subgroups. (**A**), The relationship between PFS and radiotherapy across subgroups in the entire treatment cohort; (**B**), The relationship between PFS and irAEs in the IT-RT subgroup; (**C**), The relationship between PFS and irAEs in the IT-only subgroup.

IT-RT subgroup patients: mPFS of 12.06 months, mean PFS of 13.36 months, and a one-year PFS rate of 50.00%. IT-only subgroup patients: mPFS of 11.07 months, mean PFS of 13.19 months, and a one-year PFS rate of 46.34%. Compared to the IT-only group, the IT-RT group showed no significant statistical difference in average clinical stage (*p* = 0.067). PFS curves, based on the relationship between PFS and radiotherapy in the entire treatment cohort, were plotted (Fig. 2), indicating no significant improvement in PFS for the IT-RT group compared to the IT-only group (*p* > 0.05). As shown in Table S8, there is no significant statistical difference in recent clinical efficacy evaluations between the IT-RT and IT-only groups (*p* > 0.05). 

IT-RT group (Stage IV) patients: mPFS of 10.73 months, mean PFS of 12.02 months, and a one-year PFS rate of 41.67%. IT-only group (Stage IV) patients: mPFS of 9.90 months, mean PFS of 12.36 months, and a one-year PFS rate of 39.74%. As indicated in Table S9, there is no significant statistical difference in recent clinical efficacy evaluations between the IT-RT group (Stage IV) and the IT-only group (Stage IV) (*p* > 0.05). 

In the IT-RT subgroup, patients with irAEs: mPFS of 10.87 months, mean PFS of 14.12 months, and a one-year PFS rate of 43.48%. Patients without irAEs: mPFS of 13.37 months, mean PFS of 12.65 months, and a one-year PFS rate of 56.52%. As shown in Table S10, within the IT-RT subgroup, patients without irAEs compared to those with irAEs exhibit no significant statistical difference in average clinical stage (*p* = 0.074) or in recent clinical efficacy evaluations (*p* > 0.05). PFS curves based on the relationship between PFS and irAEs in the IT-RT subgroup (Fig. 2) indicate no significant improvement in PFS for patients without irAEs compared to those with irAEs (*p* > 0.05). 

In the IT-RT subgroup, Stage IV patients with irAEs: mPFS of 9.90 months, mean PFS of 12.86 months, and a one-year PFS rate of 38.10%. Patients without irAEs: mPFS of 11.10 months, mean PFS of 10.85 months, and a one-year PFS rate of 46.67%. As indicated in Table S11, there is no significant statistical difference in recent clinical efficacy evaluations between Stage IV patients with irAEs and those without irAEs in the IT-RT subgroup (*p* > 0.05). 

In the IT-only subgroup, patients with irAEs had a median PFS of 9.53 months, a mean PFS of 11.89 months, and a one-year progression-free survival rate of 38.60%. In contrast, patients without irAEs had a median PFS of 12.58 months, a mean PFS of 14.32 months, and a one-year PFS rate of 53.03%. According to Table S12 and Fig. 2, while there were no significant statistical differences between the groups in terms of average clinical staging and recent efficacy evaluation (*p* > 0.05), patients without irAEs exhibited a significantly longer PFS (*p* = 0.012). Table S13 shows that, within the IT-only subgroup, there were no significant statistical differences in recent clinical efficacy evaluation between Stage IV patients with and without irAEs (*p* > 0.05). 

## 4 Discussion

In this study, we observed that irAEs in patients with advanced NSCLC undergoing anti-PD-1 therapy mainly manifested as dermatological, gastrointestinal, pulmonary, and endocrine toxicities, consistent with the ASCO Clinical Practice Guidelines [18]. Among these, irAE dermatitis was the most common ≥Grade 3 irAE. The occurrence of irAEs not only impacts patients’ quality of life but is also correlated with immunotherapeutic efficacy [19]. Interestingly, our findings echo previous studies, indicating that in the context of first-line anti-PD-1 therapy for advanced NSCLC, the absence of irAEs in patient cohorts is associated with favorable immunotherapy outcomes. To minimize the incidence of severe irAEs, optimize therapeutic efficacy, and enhance quality of life for patients with advanced NSCLC, our study underscores the importance of meticulous irAE risk assessment and management prior to initiating anti-PD-1 therapy. However, other studies have shown that irAEs induced by ICIs may be associated with better prognosis in NSCLC patients [20,21]. In lung cancer patients, gastrointestinal toxicities associated with irAEs may be linked to microbiota diversity [22]. Pulmonary toxicities primarily include granulomatous lung disease and pneumonia, with a notably higher incidence in overweight and obese individuals [23]. Particularly in NSCLC patients, the incidence of irAE-induced pneumonia is elevated [24], potentially related to enhanced T-cell activity and respiratory microbiome dysbiosis resulting from prolonged or extensive antibiotic use [25]. Endocrine toxicities induced by anti-PD-1 therapy predominantly affect thyroid function, typically presenting as either hyperthyroidism or hypothyroidism. Clinically, irAEs are predominantly Grade I-II, with a low incidence of ≥Grade III. Mild irAEs often do not require immediate clinical intervention, can be observed or symptomatically treated, and are self-limiting [26]. Despite the minor impact of mild irAEs on patients, their relationship with prognosis remains unclear, with limited research in this area. irAEs exhibit a double-edged effect: on one hand, mild irAEs may indicate higher response rates and better therapeutic outcomes of immune checkpoint blockade (ICB); on the other hand, severe irAEs, while potentially indicative of improved efficacy, also suggest a higher risk of adverse reactions, even life-threatening ones. Therefore, irAE management strategies should aim to identify and effectively handle mild to moderate irAEs early while preventing the occurrence of severe irAEs, thus balancing the delicate relationship between treatment efficacy and patient safety. 

Studies have demonstrated that systemic inflammatory response plays a crucial role in tumorigenesis and tumor progression [27]. Neutrophils contribute to tumor proliferation and metastasis by secreting and releasing various cytokines and pro-angiogenic factors [28]. Similarly, activated platelets promote tumor immune evasion through integrins, fibrinogen, and P-selectin [29], while monocytes exert immunosuppressive effects by recruiting Tregs within the tumor microenvironment [30]. Lymphocytes, as essential components of the immune system, have low counts indicative of poor tumor prognosis [31]. Additionally, these inflammatory cells can produce and release inflammatory mediators, such as TNFα, IL-1, and IL-6, which not only directly stimulate the synthesis and release of CRP but also attract and activate other inflammatory cells, amplifying inflammatory signals [32]. Higher CRP levels are associated with lower CD4^+^ T cell levels, which are critical for antitumor immune responses [33]. Studies have also found that elevated CRP levels are positively correlated with CD8^+^ T cell and Treg infiltration [34], leading to heightened activation of effector T cells, systemic inflammatory response, and the occurrence of various irAEs. IL-6 plays an active role in both innate and adaptive immunity, such as Th cell activation, Treg inhibition, and B cell differentiation [35]. Elevated IL-6 levels in patients undergoing immunotherapy are significantly associated with an increased incidence of irAEs [36]. ICI treatment may trigger a robust immune response, leading to increased secretion of inflammatory cytokines, recruiting more inflammatory cells to the tumor site, and amplifying the inflammatory effect [37]. PIV is a comprehensive indicator reflecting the body’s immune and inflammatory status, usually calculated by combining multiple blood parameters, namely the [(NEUT × PLT × MONO)/LYMPH] ratio. The results of this study indicate that baseline PIV levels, whether high or low, are closely associated with the occurrence of irAEs in advanced NSCLC patients before anti-PD-1 therapy. It is speculated that anti-PD-1 therapy in advanced NSCLC patients can induce an excessive immune storm, increasing the secretion of inflammatory cytokines, with high baseline PIV levels accelerating this process, ultimately leading to a significantly higher incidence of irAEs of all grades or ≥Grade III irAEs. Therefore, based on the experimental results, we hypothesize that low baseline PIV levels may be associated with a favorable prognosis in immunotherapy. 

As early as December 2018, the combination of atezolizumab, bevacizumab, paclitaxel, and carboplatin was approved by the U.S. Food and Drug Administration (FDA) and recommended as a first-line treatment for metastatic non-squamous non-small cell lung cancer (nsqNSCLC). In this study, we observed that, compared to Group A, patients in Group C had a significantly later average clinical stage (*p* = 0.001), indicating a more advanced disease progression in Group C. However, there was no significant statistical difference in Objective Response Rate (ORR) between the two groups (*p* = 0.506), suggesting that both treatment strategies may have similar efficacy in reducing tumor size. Notably, the mean PFS (13.60 months vs. 13.17 months) and one-year PFS rate (60.47% vs. 47.17%) were higher in Group C than in Group A. These results highlight that the addition of bevacizumab to a PD-1 inhibitor combined with platinum-based doublet chemotherapy may partially improve the prognosis of patients with advanced NSCLC, particularly those with Stage IV disease. The potential mechanisms for this efficacy are as follows: First, bevacizumab effectively inhibits angiogenesis by targeting VEGF, helping to restore normal vascular structure, improve blood circulation, increase tissue perfusion, and enhance T cell infiltration into tumors, thereby improving the immune microenvironment. Second, vascular normalization may enhance T cell activation and tumor antigen presentation by activating dendritic cells (DCs) [38]. Additionally, PD-1 inhibitors may induce the normalization of aberrant tumor vasculature [39]. Finally, bevacizumab increases vascular permeability and reduces interstitial pressure, thereby accelerating the delivery of cytotoxic chemotherapy drugs, increasing their concentration within tumor tissues, and ultimately enhancing the cytotoxic effect on tumor cells [40]. 

The results of the KEYNOTE-021 study indicate that compared to the combination of pembrolizumab with platinum-based chemotherapy, the addition of bevacizumab to this regimen may increase the risk of specific irAEs [41]. Similarly, our study found that introducing bevacizumab to PD-1 inhibitor-based chemotherapy could partially improve the prognosis of patients with advanced NSCLC, while also increasing the risk of irAE-related oral mucositis (*p* = 0.01). We attribute this phenomenon to several factors: First, PD-1 inhibitors activate the immune system to target tumor cells, but they may also mistakenly attack normal tissues, leading to immune-mediated tissue damage such as oral mucositis. Second, as a VEGF monoclonal antibody, bevacizumab inhibits tumor angiogenesis but also reduces blood supply to normal tissues, thereby impairing tissue self-repair. Finally, the combination of PD-1 inhibitors and bevacizumab may have a synergistic effect, enhancing anti-tumor activity but also potentially exacerbating damage to normal tissues, particularly those sensitive to injury, such as the oral mucosa. 

The results of this study indicate that combining radiotherapy with immunotherapy only increased the risk of irAE-related leukopenia, without significantly affecting other irAEs. This phenomenon may be attributed to several factors: First, radiotherapy can damage bone marrow hematopoietic stem cells, reducing leukocyte production; second, the activation and regulation effects of immunotherapy on the immune system, when combined with radiotherapy, may exacerbate the risk of leukopenia. Furthermore, during the study, a “distant effect” was observed in non-irradiated tumor sites, suggesting that radiotherapy might synergize with PD-1 inhibitors by promoting this effect, thereby enhancing the body’s immune response to tumors [42,43]. Combining radiotherapy with anti-PD-1 therapy has become a routine strategy in the comprehensive treatment of advanced NSCLC. In this study, we observed that the average clinical stage of patients in the IT-RT subgroup was later than that of the IT-only subgroup, although the difference was not statistically significant (*p* = 0.067). Notably, patients in the IT-RT subgroup had higher mPFS, mean PFS, and one-year progression-free survival rates compared to the IT-only subgroup. These findings further confirm that radiotherapy can partially enhance the efficacy of first-line anti-PD-1 therapy in patients with stage IV NSCLC, with overall safety remaining manageable. 

Albumin levels serve not only as a critical indicator of nutritional status but also as a marker of improving immune function and resilience, with elevated levels often reflecting enhanced physiological defense mechanisms [44,45]. Studies have shown that serum ALB levels are inhibited by various tumor-related cytokines and negatively correlate with tumor-induced inflammatory responses [46]. Plasma fibrinogen, a glycoprotein produced by hepatocytes, is closely associated with tumor cell dissemination and metastasis. Research indicates that FIB forms a protective layer around tumors, shielding them from NK cell-mediated cytotoxicity, and serves as a carrier for VEGF and fibroblast growth factor-2, thereby promoting tumor growth and angiogenesis [47,48]. Plasma fibrinogen interacts with specific receptors on the surface of inflammatory cells, enhancing their adhesion and migration, and activating inflammatory cells, including the promotion of NEUT and MONO production and the release of inflammatory mediators. These mediators further regulate inflammatory responses and immune reactions. Low ALB levels are commonly observed in various disease states, including tumors, reflecting the patient’s overall health status and potentially correlating with increased risks of immunotherapy tolerance and adverse reactions. Our experimental results indicate that baseline AFR levels in patients with advanced NSCLC are significantly associated with the occurrence of irAEs at all grades (*p* < 0.05). We hypothesize that low baseline AFR levels are not only closely related to the occurrence of irAEs but may also be associated with poor prognosis in immunotherapy. 

This study observed a significantly higher incidence of oral mucositis in Group C. However, it is important to emphasize that accurately attributing adverse events in combination treatment regimens is challenging. Oral mucositis may arise from multiple mechanisms: immune attack on oral epithelium by T cells activated by PD-1 inhibitors (true irAE), mucosal barrier dysfunction resulting from bevacizumab-induced inhibition of vascular endothelial repair (targeted therapy toxicity), direct damage to rapidly dividing epithelial cells by platinum agents (chemotherapy toxicity), or synergistic effects of multiple mechanisms. In this retrospective study, we lack definitive evidence to differentiate these mechanisms, such as oral mucosal biopsy pathology, local inflammatory cytokine profiles, or response patterns to specific interventions (e.g., corticosteroids vs. supportive care). Therefore, the ‘oral mucositis’ reported in our study should be understood as a clinical event rather than a mechanistically confirmed irAE. Future prospective studies should systematically collect biospecimens and clinical data that facilitate mechanistic differentiation, and consider employing adverse event attribution scoring systems (e.g., Naranjo score) to improve attribution accuracy.

Several limitations should be considered when interpreting our findings. The identified biomarkers in this study, particularly the PIV cut-off value of 500.437, lack external validation. The high AUC of 0.915, while promising, also raises concerns about potential overfitting due to the limited sample size, the low number of events, and the data-driven cut-off selection. This value should be considered hypothesis-generating rather than a clinically validated conclusion. Future studies based on independent cohorts and using pre-specified thresholds are needed. The retrospective design and relatively small sample size from a single center may introduce selection bias. Second, heterogeneity in pathological types, chemotherapy regimens, and specific PD-1 inhibitors across treatment groups could potentially influence both efficacy outcomes and adverse event profiles. Although all patients received platinum-based doublet chemotherapy as the backbone regimen, the use of different PD-1 inhibitors and chemotherapy agents based on pathological type and clinical practice may have introduced confounding variables. Future prospective studies with standardized treatment protocols are warranted to validate our findings and minimize such heterogeneity. We also acknowledge the lack of detailed subclassification for stage III patients (IIIA, IIIB, IIIC) as a limitation. Since prognosis and treatment strategies can differ among these subgroups, the absence of this granularity may have introduced heterogeneity and potentially influenced the PFS analysis. Future prospective studies with standardized staging and larger sample sizes are needed to validate our findings across specific stage III subcategories. The primary methodological limitation of this study is the use of two overlapping classification systems (based on systemic therapy and local radiotherapy exposure) for analysis without integrating them into a unified multivariable model, leading to potential confounding. The ideal analysis would have incorporated treatment group and radiotherapy status as covariates; however, complex modeling risked instability due to limited sample size and number of events. Therefore, all subgroup findings should be considered exploratory, and future prospective studies are needed to validate the independent contributions of each treatment modality. Additionally, this study did not assess whether baseline inflammatory markers, especially PIV, independently predict PFS or OS. Subgroup PFS comparisons were exploratory and lacked multivariate adjustment for confounders. Thus, no conclusions on their prognostic value for survival can be drawn. Future studies should include comprehensive survival analyses to address this question. 

## 5 Conclusion

Baseline PIV is an independent predictor of irAEs in advanced NSCLC patients receiving first-line PD-1 inhibitors. Adding bevacizumab improves efficacy in stage IV patients but increases the risk of oral mucositis. Radiotherapy moderately enhances efficacy without significantly raising overall toxicity, though leukopenia incidence rises. The absence of irAEs was associated with better PFS in the immunotherapy-only subgroup, highlighting the complex efficacy-toxicity relationship. Individualized risk stratification based on inflammatory markers is recommended.

## Data Availability

The raw data of this study will be made available by the corresponding author, without undue reservation.

## Supplementary Materials

The supplementary material is available online at https://www.techscience.com/doi/10.32604/or.2026.079813/s1.

## References

[1] WHO News Release . Global cancer burden growing, amidst mounting need for services. Saudi Med J. 2024; 45( 3): 326327. doi:10.15537/1658-3175.8333. PMC11115397 38438207

[2] Ozkarafakili MA , Yalcinkaya Kara ZM , Musluman AM , Bek TT . The association of plasma asymmetric dimethylarginine concentrations and inflammation markers in non-small cell lung cancer. Med Bull Sisli Etfal Hosp. 2024; 58( 4): 460– 7. doi:10.14744/semb.2024.29939. PMC11729830 39816429

[3] Sun X , Men Y , Hui ZG . Research status of neoadjuvant therapy for stage IIIA-N2 non-small cell lung cancer. Chin J Radiat Oncol. 2020; 29( 1): 61– 4. (In Chinese).

[4] Liu Y , Gao Y , Huo C , Zeng T , Meng W , Zhang H , et al. CAR-T therapy in non-small cell lung cancer: clinical prospects, potential, and strategies for cardiotoxicity management. Transl Oncol. 2026; 64: 102662. doi:10.1016/j.tranon.2025.102662. 41496418 PMC12813147

[5] Zhang R , Yang Q , Chen Z , Huang J , Zhang G . Synergistic antitumor effects of *Astragalus* polysaccharide: a preclinical systematic review and meta-analysis. Front Pharmacol. 2025; 16: 1672450. doi:10.3389/fphar.2025.1672450. 41487528 PMC12756362

[6] Yeong J , Suteja L , Simoni Y , Lau KW , Tan AC , Li HH , et al. Intratumoral CD39^+^CD8^+^ T cells predict response to programmed cell death protein-1 or programmed death ligand-1 blockade in patients with NSCLC. J Thorac Oncol. 2021; 16( 8): 1349– 58. doi:10.1016/j.jtho.2021.04.016. 33975004

[7] Cozma A , Sporis ND , Lazar AL , Buruiana A , Ganea AM , Malinescu TV , et al. Cardiac toxicity associated with immune checkpoint inhibitors: a systematic review. Int J Mol Sci. 2022; 23( 18): 10948. doi:10.3390/ijms231810948. 36142866 PMC9502843

[8] Gan J , Lei K , Chang T , Wang J , Yang R , Kong Q , et al. Immunotherapy with programmed cell death 1 versus programmed cell death ligand 1 inhibitors in patients with advanced non–small cell lung cancers: a multicenter, retrospective analysis. MedComm. 2025; 6( 12): e70476. doi:10.1002/mco2.70476. 41245885 PMC12618853

[9] Chen Y , Shi Y , Ding H , Feng Y , Zhang T , Liang Y , et al. Different associations between organ-specific immune-related adverse event and survival in non-small cell lung cancer patients treated with programmed death-1 inhibitors-based combination therapy. Ther Adv Med Oncol. 2023; 15: 17588359231210678. doi:10.1177/17588359231210678. 38028145 PMC10644755

[10] Liu W , Liu Y , Ma F , Sun B , Wang Y , Luo J , et al. Peripheral blood markers associated with immune-related adverse effects in patients who had advanced non-small cell lung cancer treated with PD-1 inhibitors. Cancer Manag Res. 2021; 13: 765– 71. doi:10.2147/cmar.s293200. 33536784 PMC7850423

[11] Schneider BJ , Naidoo J , Santomasso BD , Lacchetti C , Adkins S , Anadkat M , et al. Management of immune-related adverse events in patients treated with immune checkpoint inhibitor therapy: ASCO guideline update. J Clin Oncol. 2021; 39( 36): 4073– 126. doi:10.1200/JCO.21.01440. 34724392

[12] Liu Y , Meng W , Pan L , Zhang H , He Q . Molecular mechanisms and efficacy of tislelizumab, a PD-1 inhibitor developed in China, in non-small cell lung cancer. Chem Biol Drug Des. 2026; 107( 2): e70252. doi:10.1111/cbdd.70252. 41606434

[13] Singh N , Baby D , Rajguru J , Patil P , Thakkannavar S , Pujari V . Inflammation and cancer. Ann Afr Med. 2019; 18( 3): 121. doi:10.4103/aam.aam_56_18. 31417011 PMC6704802

[14] Nakayama T , Saito K , Kumagai J , Nakajima Y , Kijima T , Yoshida S , et al. Higher serum C-reactive protein level represents the immunosuppressive tumor microenvironment in patients with clear cell renal cell carcinoma. Clin Genitourin Cancer. 2018; 16( 6): e1151– 8. doi:10.1016/j.clgc.2018.07.027. 30213543

[15] Liang H , Xu Y , Chen M , Zhong W , Wang M , Zhao J . Patterns of response in metastatic NSCLC during PD-1 or PD-L1 inhibitor therapy: comparison of the RECIST 1.1 and iRECIST criteria. Thorac Cancer. 2020; 11( 4): 1068– 75. doi:10.1111/1759-7714.13367. 32129934 PMC7113040

[16] Freites-Martinez A , Santana N , Arias-Santiago S , Viera A . Using the common terminology criteria for adverse events (CTCAE–version 5.0) to evaluate the severity of adverse events of anticancer therapies. Actas Dermo Sifiliográficas Engl Ed. 2021; 112( 1): 90– 2. doi:10.1016/j.adengl.2019.05.021. 32891586

[17] Naranjo CA , Busto U , Sellers EM , Sandor P , Ruiz I , Roberts EA , et al. A method for estimating the probability of adverse drug reactions. Clin Pharmacol Ther. 1981; 30( 2): 239– 45. doi:10.1038/clpt.1981.154. 7249508

[18] Trommer M , Adams A , Celik E , Fan J , Funken D , Herter JM , et al. Oncologic outcome and immune responses of radiotherapy with anti-PD-1 treatment for brain metastases regarding timing and benefiting subgroups. Cancers. 2022; 14( 5): 1240. doi:10.3390/cancers14051240. 35267546 PMC8909717

[19] Meanwatthana J , Chantarasap P , Chuatrisorn I , Wiriya T , Jitawatanarat P . Pharmacist’s role in immune-related adverse events management: real-world incidence and risk evaluation from immunotherapy. Int J Pharm Pract. 2022; 30( 4): 377– 82. doi:10.1093/ijpp/riac048. 35731644

[20] Ma S , Nie H , Wei C , Jin C , Wang L . Association between immune-related adverse events and prognosis in patients with advanced non-small cell lung cancer: a systematic review and meta-analysis. Front Oncol. 2024; 14: 1402017. doi:10.3389/fonc.2024.1402017. 38779082 PMC11109391

[21] Lin L , Liu Y , Chen C , Wei A , Li W . Association between immune-related adverse events and immunotherapy efficacy in non-small-cell lung cancer: a meta-analysis. Front Pharmacol. 2023; 14: 1190001. doi:10.3389/fphar.2023.1190001. 37284302 PMC10239972

[22] Chau J , Yadav M , Liu B , Furqan M , Dai Q , Shahi S , et al. Prospective correlation between the patient microbiome with response to and development of immune-mediated adverse effects to immunotherapy in lung cancer. BMC Cancer. 2021; 21( 1): 808. doi:10.1186/s12885-021-08530-z. 34256732 PMC8278634

[23] Eun Y , Kim IY , Sun JM , Lee J , Cha HS , Koh EM , et al. Risk factors for immune-related adverse events associated with anti-PD-1 pembrolizumab. Sci Rep. 2019; 9( 1): 14039. doi:10.1038/s41598-019-50574-6. 31575933 PMC6773778

[24] Zhang Q , Tang L , Zhou Y , He W , Li W . Immune checkpoint inhibitor-associated pneumonitis in non-small cell lung cancer: current understanding in characteristics, diagnosis, and management. Front Immunol. 2021; 12: 663986. doi:10.3389/fimmu.2021.663986. 34122422 PMC8195248

[25] Jing Y , Chen X , Li K , Liu Y , Zhang Z , Chen Y , et al. Association of antibiotic treatment with immune-related adverse events in patients with cancer receiving immunotherapy. J Immunother Cancer. 2022; 10( 1): e003779. doi:10.1136/jitc-2021-003779. 35058327 PMC8772460

[26] Wang DY , Salem JE , Cohen JV , Chandra S , Menzer C , Ye F , et al. Fatal toxic effects associated with immune checkpoint inhibitors: a systematic review and meta-analysis. JAMA Oncol. 2018; 4( 12): 1721. doi:10.1001/jamaoncol.2018.3923. 30242316 PMC6440712

[27] Bi Q , Wu JY , Qiu XM , Zhang JD , Sun ZJ , Wang W . Tumor-associated inflammation: the tumor-promoting immunity in the early stages of tumorigenesis. J Immunol Res. 2022; 2022: 3128933. doi:10.1155/2022/3128933. 35733919 PMC9208911

[28] Xiong S , Dong L , Cheng L . Neutrophils in cancer carcinogenesis and metastasis. J Hematol Oncol. 2021; 14( 1): 173. doi:10.1186/s13045-021-01187-y. 34674757 PMC8529570

[29] Gan J , Zhang X , Guo J . The role of platelets in tumor immune evasion and metastasis: mechanisms and therapeutic implications. Cancer Cell Int. 2025; 25( 1): 258. doi:10.1186/s12935-025-03877-w. 40646579 PMC12255057

[30] Olingy CE , Dinh HQ , Hedrick CC . Monocyte heterogeneity and functions in cancer. J Leukoc Biol. 2019; 106( 2): 309– 22. doi:10.1002/jlb.4ri0818-311r. 30776148 PMC6658332

[31] Ueda K , Suekane S , Kurose H , Ogasawara N , Hiroshige T , Chikui K , et al. Absolute lymphocyte count is an independent predictor of survival in patients with metastatic renal cell carcinoma treated with nivolumab. Jpn J Clin Oncol. 2022; 52( 2): 179– 86. doi:10.1093/jjco/hyab157. 34607361

[32] Ravindranathan D , Master VA , Bilen MA . Inflammatory markers in cancer immunotherapy. Biology. 2021; 10( 4): 325. doi:10.3390/biology10040325. 33924623 PMC8069970

[33] Iivanainen S , Ahvonen J , Knuuttila A , Tiainen S , Koivunen JP . Elevated CRP levels indicate poor progression-free and overall survival on cancer patients treated with PD-1 inhibitors. ESMO Open. 2019; 4( 4): e000531. doi:10.1136/esmoopen-2019-000531. 31555483 PMC6735669

[34] Roser LA , Luckhardt S , Ziegler N , Thomas D , Wagner PV , Damm G , et al. Immuno-inflammatory *in vitro* hepatotoxicity models to assess side effects of biologicals exemplified by aldesleukin. Front Immunol. 2023; 14: 1275368. doi:10.3389/fimmu.2023.1275368. 38045689 PMC10693457

[35] Wangriatisak K , Kochayoo P , Thawornpan P , Leepiyasakulchai C , Suangtamai T , Ngamjanyaporn P , et al. CD4^+^ T-cell cooperation promoted pathogenic function of activated naïve B cells of patients with SLE. Lupus Sci Med. 2022; 9( 1): e000739. doi:10.1136/lupus-2022-000739. 36180106 PMC9528597

[36] Ma H , Zhang S , Jiao P , Ding H , Wang F , Zhao Y , et al. Serum IL-6 predicts immunotherapy-related adverse and outcome in advanced gastric and esophageal cancer patients with Anti-PD-1 treatment. Front Immunol. 2025; 16: 1553882. doi:10.3389/fimmu.2025.1553882. 40519900 PMC12163003

[37] Zarifa A , Kim JW , Lopez-Mattei J , Palaskas N , Iliescu C , Kim PY . Cardiac toxicities associated with immune checkpoints inhibitors: mechanisms, manifestations and management. Korean Circ J. 2021; 51( 7): 579– 97. doi:10.4070/kcj.2021.0089. 34227272 PMC8263294

[38] Fukumura D , Kloepper J , Amoozgar Z , Dan DG , Jain RK . Enhancing cancer immunotherapy using antiangiogenics: opportunities and challenges. Nat Rev Clin Oncol. 2018; 15( 5): 325– 40. doi:10.1038/nrclinonc.2018.29. 29508855 PMC5921900

[39] Hao S , Ai D , Wang Q , Zhang X , Ma X , Tseng I , et al. PD-1 inhibitor improves radiosensitivity by tumor vessel normalization. Br J Cancer. 2026; 134( 5): 820– 30. doi:10.1038/s41416-025-03315-8. 41454187 PMC12905340

[40] Majidpoor J , Mortezaee K . Angiogenesis as a hallmark of solid tumors—clinical perspectives. Cell Oncol. 2021; 44( 4): 715– 37. doi:10.1007/s13402-021-00602-3. PMC12980750 33835425

[41] Awad MM , Gadgeel SM , Borghaei H , Patnaik A , Yang JC , Powell SF , et al. Long-term overall survival from KEYNOTE-021 cohort G: pemetrexed and carboplatin with or without pembrolizumab as first-line therapy for advanced nonsquamous NSCLC. J Thorac Oncol. 2021; 16( 1): 162– 8. doi:10.1016/j.jtho.2020.09.015. 33069888

[42] Huang J , Theelen WSME , Belcaid Z , Najjar M , van der Geest D , Singh D , et al. Combination of pembrolizumab and radiotherapy induces systemic antitumor immune responses in immunologically cold non-small cell lung cancer. Nat Cancer. 2025; 6( 10): 1676– 92. doi:10.1038/s43018-025-01018-w. 40696153 PMC12559004

[43] Meng W , Zhu Y , Wang J , Gan J , Liu J , Wang D , et al. Intra-arterial delivery of tislelizumab plus transarterial chemoembolization for rectal cancer: a novel regimen to achieve sphincter preservation and prevent anastomotic leakage. MedComm. 2025; 6( 11): e70456. doi:10.1002/mco2.70456. 41164798 PMC12559920

[44] Fan Y , Xiang S , Dai Z , Zou C , Wang X , Gao Z . Prognostic significance of C-reactive protein to albumin ratio in colorectal cancer patients: a meta-analysis. Int J Colorectal Dis. 2019; 34( 6): 1105– 11. doi:10.1007/s00384-019-03299-x. 31016379

[45] Pinato DJ , Sharma R , Citti C , Platt H , Ventura-Cots M , Allara E , et al. The albumin-bilirubin grade uncovers the prognostic relationship between hepatic reserve and immune dysfunction in HIV-associated hepatocellular carcinoma. Aliment Pharmacol Ther. 2018; 47( 1): 95– 103. doi:10.1111/apt.14356. 29034998

[46] Rong Z , Liu J , Cheng W , Liu Y , Duan Y , Hu A , et al. Correlation between the prognostic nutritional index and breast cancer in U.S. adults: NHANES 2001-2018. Nutr Cancer. 2025; 77( 10): 1162– 72. doi:10.1080/01635581.2025.2559436. 40963229

[47] Hou C , Jiang F , Ma H , Zhu Q , Wang Z , Zhao B , et al. Prognostic role of preoperative platelet, fibrinogen, and D-dimer levels in patients with non-small cell lung cancer: a multicenter prospective study. Thorac Cancer. 2019; 10( 2): 304– 11. doi:10.1111/1759-7714.12956. 30609303 PMC6360242

[48] Laurent-Issartel C , Landras A , Agniel R , Giffard F , Blanc-Fournier C , Da Silva Cruz E , et al. Ascites microenvironment conditions the peritoneal pre-metastatic niche to promote the implantation of ovarian tumor spheroids: involvement of fibrinogen/fibrin and αV and α5β1 integrins. Exp Cell Res. 2024; 441( 1): 114155. doi:10.1016/j.yexcr.2024.114155. 39002689

