# Supplementary material for: Factors Affecting the Efficacy and Safety of First-Line Anti-PD-1 Therapy in Advanced Non-Small Cell Lung Cancer
Source: Oncol Res. 2026 Jun 16;34(7):27. doi: 10.32604/or.2026.079813 (PMC13292038; doi:10.32604/or.2026.079813)
Supplement: Supplementary file 1 [file OncolRes-34-79813-s001.zip › TSP_OR_79813-s001.docx]

Supplementary Material

S1 Study Subjects and Methods

S1.1 Inclusion Criteria

1. Based on the eighth edition of the TNM staging system by the International Union Against Cancer (UICC), all patients were classified as having stage III or IV lung cancer; (2) Patients must have received at least three cycles of first-line therapy primarily consisting of PD-1 inhibitors; (3) Imaging assessments were conducted every 2-3 cycles to evaluate clinical treatment efficacy; (4) Patients had at least one measurable target lesion according to RECIST 1.1 criteria; (5) Patients had not undergone prolonged glucocorticoid therapy; (6) All patients had a Karnofsky Performance Status (KPS) score of ≥60; (7) Physiological functions were generally normal, with peripheral blood cell counts meeting the criteria for anticancer drug treatment. Total bilirubin was ≤2.0 times the upper limit of normal (ULN), aspartate aminotransferase and alanine aminotransferase were ≤3 times ULN, and serum creatinine was ≤1.6 times ULN.

S1.2 Exclusion Criteria

(1) Pulmonary metastatic tumors or previously existing malignancies in other locations (n=2,646); (2) Concurrent acute infectious diseases, acute myocardial infarction, or severe hepatic and renal diseases (n=421); (3) Severe immune system diseases or hematologic disorders (n=238); (4) Intolerance or allergic reactions to the study drugs (n=165); (5) Recent anticoagulant therapy or major surgical procedures within the past month (n=300); (6) Untreated or unstable active brain metastases (n=200); (7) Patients lost to follow-up (n=110). (8) Patients on long-term corticosteroids at baseline were excluded during data screening (n=201).

S1.3 Laboratory Tests

All blood tests were conducted using disposable human venous blood sample collection containers. The specifications and configurations are as follows: complete blood count (EDTA-K2 2 mL, configuration: K2E-020PXZGD01-10), liver and kidney function tests, and tumor marker tests (clot activator 5 mL, configuration: SCA-050PXRCG03-00), coagulation tests (sodium citrate 1:9 2 mL, configuration: 9NC020PXLGD01-10). These materials were procured from Weihai Wego Blood Collection Products Co., Ltd. (Shandong, China).

Blood samples were collected in the early morning while patients were in a resting state. For the complete blood count and coagulation tests, 2 mL of fasting venous blood was collected, and for liver, kidney function, and tumor marker tests, 3 mL of fasting venous blood was collected. The samples were analyzed using a Sysmex XN-2800 fully automated hematology analyzer (Sysmex corporation, Kobe, Hyogo, Japan), ensuring the instrument was in optimal working condition as per the standard operating procedures (SOP) for routine blood tests and clinical trial protocols. All blood samples were tested within 3 hours of collection.

S1.4 Experimental Drugs

S1.4.1 PD-1 Inhibitors

The PD-1 inhibitors used in this study for all enrolled patients are as follows: (1) Pembrolizumab (Merck Sharp & Dohme (China) Ltd., SJ20180019, Shanghai, China); (2) Tislelizumab (Guangzhou BeiGene Biopharmaceutical Co., Ltd., S20190045, Guangzhou, Guangdong, China); (3) Camrelizumab (Suzhou Sinovent Pharmaceuticals Co., Ltd., S20190027, Suzhou, Jiangsu, China); (4) Toripalimab (Shanghai Junshi Biosciences Co., Ltd., S20180015, Shanghai, China); (5) Serplulimab (Shanghai Henlius Biotech Co., Ltd., S20220013, Shanghai, China); (6) Sintilimab (Innovent Biologics (Suzhou) Co., Ltd., S20180016, Suzhou, Jiangsu, China); (7) Penpulimab (Akeso Biopharma Co., Ltd., S20210033, Zhongshan, Guangdong, China).

Standard dosage and administration: Toripalimab is administered at 240 mg per dose, Serplulimab at 4.5 mg/kg, and the others at 200 mg per dose. The aforementioned drugs were administered via intravenous infusion once every three cycles, with a minimum of four cycles or more.

S1.4.2 Bevacizumab

The bevacizumab used by all enrolled patients in this study falls into two categories: (1)Avastin [Manufacturer: Roche Diagnostics GmbH]; (2)Anke [Manufacturer: Qilu Pharmaceutical Co., Ltd.].

The specific treatment regimen for these drugs is as follows: bevacizumab is administered via intravenous infusion on the first day of the treatment cycle, with a standard dosage of 15 mg/kg of body weight, administered once every three weeks. All cases involve retrospective data, and due to individual differences and the unavoidable presence of TRAEs, the treatment cycle and dosage may vary among individuals.

S1.4.3 Drug Administration

All cases were based on retrospective data. Due to individual variability and the inevitability of TRAE, drug dosages were adjusted after standard dosing according to the severity of the associated adverse reactions. Consequently, not all patients received subsequent treatment following the standard drug dosage and treatment schedule.


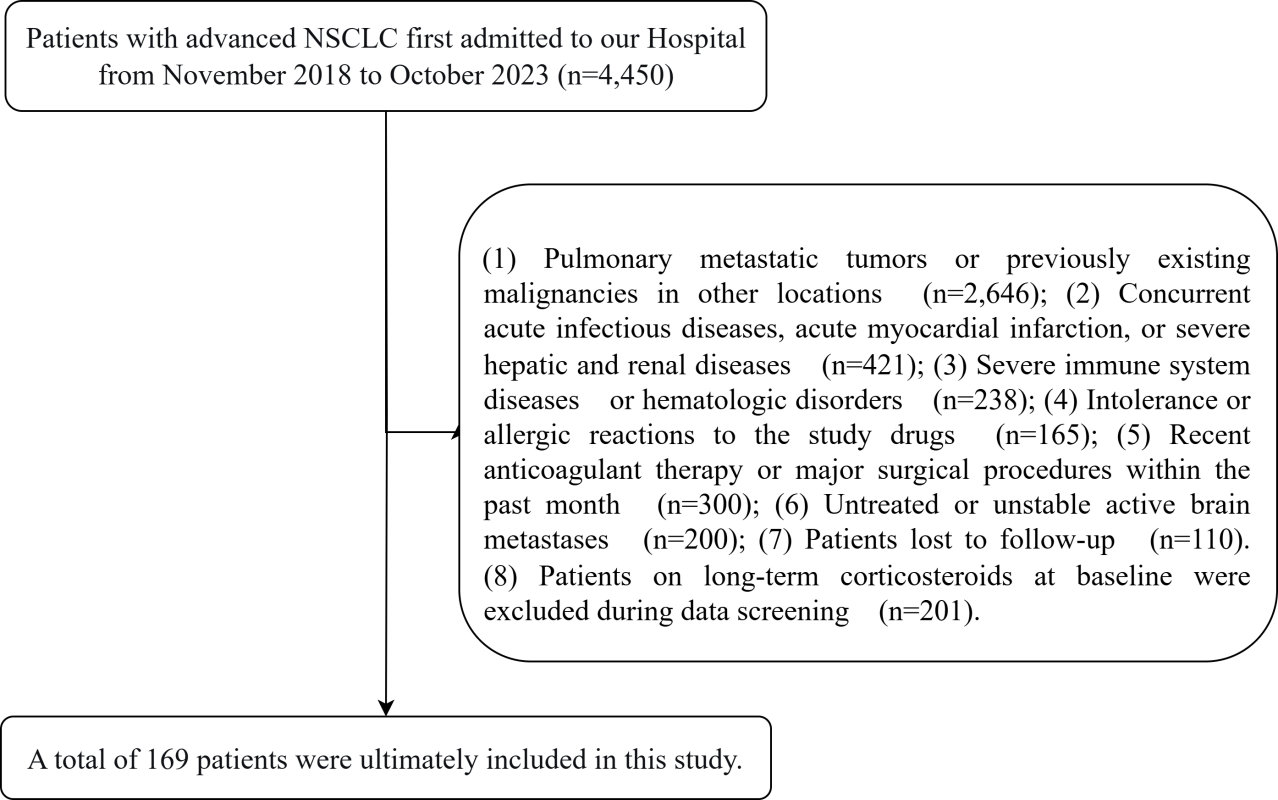


Figure S1 Patient Selection Flowchart

Table S1 Norris Drug Adverse Reaction Evaluation Criteria

| Evaluation Criteria | Issue Score | | |
| --- | --- | --- | --- |
|  | Yes | No | Unknown |
| 1. Was there a comprehensive report prior to the occurrence of this adverse reaction? | +1 | 0 | 0 |
| 1. Did this adverse reaction occur after the administration of the suspected medication? | +2 | -1 | 0 |
| 1. Did the adverse reaction improve after discontinuing the medication or using other antagonists? | +1 | 0 | 0 |
| 1. Did the adverse reaction recur upon readministration of the medication? | +2 | -1 | 0 |
| 5. Are there any other potential causes for the adverse reaction? | -1 | +2 | 0 |
| 6. Does the adverse reaction recur upon administration of a placebo? | -1 | +1 | 0 |
| 7. Is the blood drug concentration at a toxic level? | +1 | 0 | 0 |
| 8. Is the severity of the adverse reaction related to changes in the dosage of the suspected drug? | +1 | 0 | 0 |
| 9. Did the patient experience adverse reactions with this drug or similar drugs in the past? | +1 | 0 | 0 |
| 10. Has the adverse reaction been confirmed through objective examination? | +1 | 0 | 0 |

A total NCI score of ≥5 indicates a definite or highly probable relationship between the drug and the adverse reaction, supported by objective evidence or quantitative test results^[14]^.

Table S2 Incidence and Grading Statistics of irAEs in Each Patient Group

| ymptoms | Grade 1-5 Group | | | Grade≥3 Group | | | Discontinuation Group | | |
| --- | --- | --- | --- | --- | --- | --- | --- | --- | --- |
|  | A | B | C | A | B | C | A | B | C |
| Dermatitis | 20 | 3 | 3 | 8 | 0 | 2 | 5 | 0 | 1 |
| Anemia | 2 | 0 | 1 | 1 | 0 | 1 | 1 | 0 | 0 |
| Fever | 3 | 0 | 3 | 0 | 0 | 0 | 0 | 0 | 1 |
| Pneumonia | 11 | 1 | 1 | 2 | 0 | 1 | 6 | 0 | 1 |
| Fatigue | 1 | 1 | 0 | 1 | 0 | 0 | 0 | 0 | 0 |
| Leukopenia | 0 | 3 | 1 | 0 | 0 | 0 | 0 | 0 | 0 |
| Neutropenia | 0 | 4 | 0 | 0 | 0 | 0 | 0 | 0 | 0 |
| Thrombocytopenia | 1 | 0 | 1 | 0 | 0 | 1 | 0 | 0 | 0 |
| Liver dysfunction | 2 | 1 | 1 | 0 | 1 | 0 | 0 | 0 | 0 |
| Dysphagia | 0 | 2 | 0 | 0 | 0 | 0 | 0 | 0 | 0 |
| Gastrointestinal reactions | 1 | 4 | 3 | 1 | 0 | 1 | 0 | 0 | 1 |
| Blurred vision | 2 | 0 | 1 | 0 | 0 | 0 | 0 | 0 | 1 |
| Thyroid dysfunction | 11 | 1 | 4 | 0 | 0 | 0 | 1 | 0 | 0 |
| Capillary hyperplasia | 4 | 2 | 2 | 1 | 0 | 1 | 1 | 0 | 1 |
| Telangiectasia | 1 | 0 | 0 | 0 | 0 | 0 | 1 | 0 | 0 |
| Oral mucositis | 0 | 0 | 4 | 0 | 0 | 1 | 0 | 0 | 0 |
| Myocarditis | 1 | 0 | 2 | 0 | 0 | 2 | 1 | 0 | 0 |
| Total | 60 | 22 | 27 | 14 | 1 | 10 | 16 | 0 | 6 |

Table S3 ROC tables evaluating various grades of irAEs based on inflammatory markers

| Variables | Area | Standard Error | Asymptotic Significance | Asymptotic 95% Confidence Interval | |
| --- | --- | --- | --- | --- | --- |
|  |  |  |  | upper limit | lower limit |
| AFR | 0.710 | 0.040 | 0.047^*^ | 0.636 | 0.777 |
| CAR | 0.510 | 0.045 | 0.833 | 0.432 | 0.587 |
| PIV | 0.915 | 0.022 | < 0.01^**^ | 0.863 | 0.953 |
| NLR | 0.511 | 0.045 | 0.813 | 0.433 | 0.588 |
| PLR | 0.545 | 0.045 | 0.317 | 0.466 | 0.621 |
| LMR | 0.516 | 0.045 | 0.728 | 0.438 | 0.593 |

* *P*<0.05, ***P*<0.01. ROC, Receiver Operating Characteristic. AFR, ALB/FIB, albumin/fibrinogen ratio; CAR, CRP/ALB, C-reactive protein/albumin ratio; PIV, (NEUT×PLT×MONO)/LYMPH, (neutrophils×platelets×monocytes) / lymphocytes ratio; NLR, NEUT/LYMPH, neutrophils/lymphocytes ratio; PLR, PLT/LYMPH, platelets/lymphocytes ratio; LMR, LYMPH/MONO, lymphocytes/monocytes ratio.

Table S4 ROC tables evaluating grade≥3 irAEs based on inflammatory markers

| Variables | Area | Standard Error | Asymptotic Significance | Asymptotic 95% Confidence Interval | |
| --- | --- | --- | --- | --- | --- |
|  |  |  |  | upper limit | lower limit |
| AFR | 0.577 | 0.063 | 0.931 | 0.498 | 0.652 |
| CAR | 0.510 | 0.072 | 0.862 | 0.432 | 0.588 |
| PIV | 0.719 | 0.050 | < 0.001^***^ | 0.645 | 0.785 |
| NLR | 0.510 | 0.075 | 0.883 | 0.432 | 0.587 |
| PLR | 0.530 | 0.066 | 0.647 | 0.452 | 0.607 |
| LMR | 0.548 | 0.067 | 0.463 | 0.469 | 0.624 |

*** P<0.001.

Table S5 Comparison of the incidence of common irAEs between Group A and Group C patient

| Adverse reactions | Group A | Group C | Statistical values | *p*-value |
| --- | --- | --- | --- | --- |
|  | (n=106) | (n=43) |  |  |
| Dermatitis , n (%) | 20 (18.87) | 3 (6.98) | 3.314 | 0.069 |
| Thyroid dysfunction , n (%) | 11 (10.38) | 4 (9.30) | 0.039 | 0.843 |
| Pneumonia , n (%) | 11 (10.38) | 1 (2.32) | 2.678 | 0.102 |
| Capillary proliferation , n (%) | 4 (3.77) | 0 (0.00) | 0.010 | 0.986 |
| Fever , n (%) | 3 (2.83) | 3 (6.98) | 0.500 | 0.480 |
| Gastrointestinal reactions , n (%) | 0 (0.00) | 3 (6.98) | 4.262 | 0.039^*^ |
| Oral mucositis , n (%) | 0 (0.00) | 4 (9.30) | 6.591 | 0.010^*^ |

* P<0.05.

Table S6 Clinical efficacy evaluation of patients in Groups A and C

| Groups | Stage III | Stage IV | iCR | iPR | iSD | ORR | one-year PFS rate |
| --- | --- | --- | --- | --- | --- | --- | --- |
| Group A, n (%) | 43  (40.57) | 63  (59.43) | 1  (0.94) | 42  (39.62) | 63  (59.44) | 43  (40.56) | 50  (47.17) |
| Group C, n (%) | 5  (11.63) | 38  (88.37) | 0  (0.00) | 20  (46.51) | 23  (53.49) | 20  (46.51) | 19  (44.19) |
| statistical values | 11.731 | 11.731 | 0.408 | 0.598 | 0.443 | 0.443 | 0.110 |
| *p*-value | 0.001^*^ | 0.001^*^ | 0.523 | 0.44 | 0.506 | 0.506 | 0.741 |

Abbreviations: iCR, immune complete response; iPR, immune partial response; iSD, immune stable disease; ORR, objective response rate. ^*^*P*＜0.05 indicates statistical significance.

Table S7 Clinical efficacy evaluation of Group A (Stage IV) and Group C (Stage IV) patients

| Groups | iCR | iPR | iSD | ORR | one-year PFS rate |
| --- | --- | --- | --- | --- | --- |
| Group A, n (%) | 1 (1.59) | 21 (33.33) | 41 (65.08) | 22 (34.92) | 24 (38.10) |
| Group C, n (%) | 0 (0.00) | 18 (47.37) | 20 (52.63) | 18 (47.37) | 16 (42.11) |
| statistical values | - | 1.970 | 1.535 | 1.535 | 0.159 |
| *p*-value | > 0.999 | 0.160 | 0.215 | 0.215 | 0.690 |

Abbreviations:iCR, immune complete response; iPR, immune partial response; iSD, immune stable disease; ORR, objective response rate.

Table S8 Clinical efficacy evaluation of patients in the IT-RT group and the IT-only group

| Groups | Stage III | Stage IV | iCR | iPR | iSD | ORR | one-year PFS rate |
| --- | --- | --- | --- | --- | --- | --- | --- |
| IT-RT group, n (%) | 10 (21.74) | 36 (78.26) | 0 (0.00) | 17 (36.96) | 29 (63.04) | 17 (36.96) | 23 (50.00) |
| IT-only group, n (%) | 45 (36.59) | 78 (63.41) | 2 (1.63) | 50 (40.65) | 71 (57.72) | 52(42.28) | 57 (46.34) |
| statistical values | 3.361 | 3.361 | 0.757 | 0.191 | 0.392 | 0.392 | 0.180 |
| *p*-value | 0.067 | 0.067 | 0.384 | 0.662 | 0.531 | 0.531 | 0.672 |

Abbreviations:iCR, immune complete response; iPR, immune partial response; iSD, immune stable disease; ORR, objective response rate.

Table S9 Clinical efficacy evaluation of Stage IV patients in the IT-RT group and the IT-only group

| Groups | iCR | iPR | iSD | ORR | one-year PFS rate |
| --- | --- | --- | --- | --- | --- |
| IT-RT group, n (%) | 0 (0.00) | 11 (30.56) | 25 (69.44) | 11 (30.56) | 15 (41.67) |
| IT-only group, n (%) | 2 (2.56) | 31 (39.74) | 45 (57.70) | 33 (42.30) | 31 (39.74) |
| statistical values | - | 0.894 | 1.435 | 1.435 | 0.038 |
| *p*-value | > 0.999 | 0.344 | 0.231 | 0.231 | 0.846 |

Abbreviations:iCR, immune complete response; iPR, immune partial response; iSD, immune stable disease; ORR, objective response rate.

Table S10 Efficacy evaluation of patients with and without irAEs in the IT-RT subgroup

| Groups | Stage III | Stage IV | iCR | iPR | iSD | ORR | one-year PFS rate |
| --- | --- | --- | --- | --- | --- | --- | --- |
| Group with irAEs, n (%) | 2 (8.70) | 21 (91.30) | 0 (0.00) | 9 (39.13) | 14 (60.87) | 9 (39.13) | 10 (43.48) |
| Group without irAEs, n (%) | 8 (34.78) | 15 (65.22) | 0 (0.00) | 8 (34.78) | 15 (65.22) | 8 (34.78) | 13  (56.52) |
| statistical values | 3.194 | 3.194 | — | 0.093 | 0.093 | 0.093 | 0.783 |
| *p*-value | 0.074 | 0.074 | — | 0.76 | 0.76 | 0.76 | 0.376 |

Abbreviations:iCR, immune complete response; iPR, immune partial response; iSD, immune stable disease; ORR, objective response rate.

Table S11 Efficacy evaluation of Stage IV patients with and without irAEs in the IT-RT subgroup

| Groups | iCR | iPR | iSD | ORR | one-year PFS rate |
| --- | --- | --- | --- | --- | --- |
| Group with irAEs, n (%) | 0 (0.00) | 8 (38.10) | 13 (61.90) | 8 (38.10) | 8 (38.10) |
| Group without irAEs, n (%) | 0 (0.00) | 3 (20.00) | 12 (80.00) | 3 (20.00) | 7 (46.67) |
| statistical values | — | 0.632 | 0.632 | 0.632 | 0.264 |
| *p*-value | — | 0.427 | 0.427 | 0.427 | 0.607 |

Abbreviations:iCR, immune complete response; iPR, immune partial response; iSD, immune stable disease; ORR, objective response rate.

Table S12 Efficacy evaluation of patients with and without irAEs in the IT-only subgroup

| Groups | Stage III | Stage IV | iCR | iPR | iSD | ORR | one-year PFS rate |
| --- | --- | --- | --- | --- | --- | --- | --- |
| Group with irAEs, n (%) | 17 (29.82) | 40 (70.18) | 2 (3.51) | 24 (42.11) | 31 (54.38) | 26 (45.62) | 22 (38.60) |
| Group without irAEs, n (%) | 28  (42.42) | 38 (57.58) | 0 (0.00) | 26 (43.33) | 40 (56.67) | 26 (43.33) | 35 (53.03) |
| statistical values | 2.093 | 2.093 | 2.354 | 0.093 | 0.485 | 0.485 | 2.563 |
| *p*-value | 0.148 | 0.148 | 0.125 | 0.76 | 0.486 | 0.486 | 0.109 |

Abbreviations:iCR, immune complete response; iPR, immune partial response; iSD, immune stable disease; ORR, objective response rate.

Table S13 Clinical efficacy evaluation of patients with and without irAEs (Stage IV) in the IT-only subgroup

| Groups | iCR | iPR | iSD | ORR | one-year PFS rate |
| --- | --- | --- | --- | --- | --- |
| Group with irAEs, n (%) | 2 (5.00) | 17 (42.50) | 21 (52.50) | 19 (47.50) | 15 (37.50) |
| Group without irAEs, n (%) | 0 (0.00) | 14 (36.84) | 24 (63.16) | 14 (36.84) | 16 (42.11) |
| statistical values | — | 0.260 | 0.907 | 0.907 | 0.173 |
| *p*-value | 0.494 | 0.610 | 0.341 | 0.341 | 0.678 |

Abbreviations:iCR, immune complete response; iPR, immune partial response; iSD, immune stable disease; ORR, objective response rate.
